# Effect of ultrasound-guided scalp nerve block on hemodynamics and postoperative agitation in hypertensive cerebral hemorrhage craniotomy patients: a prospective randomized controlled study

**DOI:** 10.3389/fneur.2025.1721992

**Published:** 2026-01-14

**Authors:** Bing Li, Zhifang Cao, Junlong Huang, Ying Chen, Zhihua Li, Weiming Wu, Chuntian Li, Ting Qiu, Juncheng Chen

**Affiliations:** 1 Department of Anesthesiology, Longyan First Affiliated Hospital of Fujian Medical University, Longyan, China; 2 Department of Neurosurgery, Longyan First Affiliated Hospital of Fujian Medical University, Longyan, China

**Keywords:** craniotomy, dexmedetomidine, hypertensive intracerebral hemorrhage, postoperative analgesia, ropivacaine, scalp nerve block

## Abstract

**Objective:**

This study aims to investigate the impact of dexmedetomidine combined with ropivacaine scalp nerve block (SNB) on analgesic effects in patients undergoing craniotomy for hypertensive intracerebral hemorrhage (HICH).

**Methods:**

A prospective randomized controlled trial was conducted on 120 HICH patients who underwent craniotomy at Longyan First Affiliated Hospital of Fujian Medical University from May 2022 to April 2024. Patients were randomly divided into three groups: control group (A, no SNB), ropivacaine SNB group (B, 0.5% ropivacaine), and dexmedetomidine-ropivacaine SNB group (C, 0.5% ropivacaine + 0.5 μg/kg dexmedetomidine), with 40 patients in each group. Primary outcomes included intraoperative hemodynamic parameters [mean arterial pressure (MAP), heart rate (HR)], intraoperative anesthetic consumption, postoperative Visual Analogue Scale (VAS) scores, rescue analgesia requirements, and incidence of adverse reactions within 48 h postoperatively.

**Results:**

General characteristics were comparable among the three groups (*p* > 0.05). Compared with Group A, Groups B and C showed more stable intraoperative MAP and HR (*p* < 0.05), lower VAS scores at 6, 12, 24, and 48 h postoperatively (*p* < 0.01), reduced consumption of propofol and remifentanil (*p* < 0.01), delayed first rescue analgesia (*p* < 0.01), fewer rescue analgesia administrations (*p* < 0.01), and lower incidences of nausea/vomiting and postoperative agitation (*p* < 0.05). Group C exhibited superior outcomes to Group B in VAS scores (6, 12, 24, and 48 h), anesthetic consumption, and rescue analgesia metrics (*p* < 0.05), with no significant difference in adverse reactions (*p* > 0.05).

**Conclusion:**

Dexmedetomidine combined with ropivacaine for SNB improves postoperative analgesia, stabilizes intraoperative hemodynamics, reduces anesthetic usage and rescue analgesia needs, and lowers the incidence of adverse reactions in HICH craniotomy patients, making it a safe and effective analgesic strategy.

**Clinical trial registration:**

chictr.org.cn, identifier ChiCTR2500106043.

## Introduction

Hypertensive cerebral hemorrhage is a critical neurological emergency that poses significant challenges in the management of anesthesia and postoperative care. Patients with hypertensive cerebral hemorrhage often experience increased intracranial pressure, which directly affects the central nervous system, circulatory system, and respiratory function, leading to complex pathophysiological changes and life-threatening conditions (1). The maintenance of hemodynamic stability during surgery, the inhibition of adverse reflexes, the alleviation of postoperative pain, and the reduction of myocardial ischemia and the incidence of postoperative deep vein thrombosis are all crucial directions for the development of neurosurgical anesthesia.

Postoperative pain is a common and debilitating complication following craniotomy, with over 80% of patients experiencing moderate to severe pain that can persist up to 48 h post-surgery, severely impacting their daily activities (2, 3). Previous studies have indicated that postoperative pain can trigger a stress response, leading to increased sympathetic activity, cerebral blood flow, oxygen consumption, and intracranial pressure, resulting in complications such as postoperative hypertension, restlessness, nausea, vomiting, and an increased risk of intracerebral hemorrhage (3–5). Inadequate control of postoperative pain can lead to severe consequences, including agitation, intracranial hypertension, and postoperative bleeding, potentially increasing morbidity and mortality rates. Due to the side effects of opioid analgesics, there is a necessity to minimize dependence on opioids for post-craniotomy patients. A multimodal analgesic approach combining low-dose systemic analgesics with local anesthetics for scalp infiltration or regional scalp nerve blocks (SNB) has been proposed to prevent post-craniotomy pain in adults. However, the optimal postoperative pain management strategy for craniotomy patients remains inconclusive (5).

The use of ultrasound-guided SNB has been shown to improve the accuracy and efficacy of local anesthetic administration, reducing the volume of local anesthetic required and enhancing the quality of block (6). Ultrasound guidance allows for real-time visualization of the needle and the spread of local anesthetic, ensuring precise placement and potentially reducing complications associated with blind injections (7). In the context of hypertensive cerebral hemorrhage craniotomy patients, the integration of ultrasound-guided SNB with adjuvants like dexmedetomidine may offer a more effective approach to postoperative pain management, contributing to hemodynamic stability and reducing the risk of postoperative complications.

In clinical practice, SNB using ropivacaine is commonly used for postoperative analgesia in patients undergoing craniotomy. However, the duration of analgesia is often short, lasting only 3 to 5 h (6). Dexmedetomidine, a highly potent *α*_2_-adrenergic agonist with sedative, analgesic, and anti-sympathetic effects, has been widely applied as an adjuvant in regional nerve blocks (8, 9). A recent meta-analysis of randomized controlled trials provides high-certainty evidence that ropivacaine scalp block significantly reduces pain at 6 h postoperatively, while the evidence for the additive effect of adjuvants like dexmedetomidine remains inconclusive (10). This study aims to investigate the impact of dexmedetomidine combined with ropivacaine SNB on the analgesic effects in elderly patients undergoing craniotomy.

## Methods

### Study design and patient selection

This prospective, randomized controlled trial was conducted from May 2022 to April 2024 at Longyan First Affiliated Hospital of Fujian Medical University. We enrolled 120 patients with hypertensive intracerebral hemorrhage (HICH) undergoing craniotomy, aged ≥60 years, with American Society of Anesthesiologists (ASA) physical status II to IV. Inclusion criteria included: ① age≥60 years; ② ASA II-IV; ③ diagnosis confirmed by head CT or MRI according to the criteria in the “Multidisciplinary Diagnosis and Treatment Guidelines for Hypertensive Intracerebral Hemorrhage in China”; ④ first episode of disease, meeting surgical indications; ⑤ admission within 24 h after onset, with intracerebral hemorrhage volume of 30–60 mL; ⑥ patient or family fully understood the surgical and anesthesia risks and signed the informed consent form.

Exclusion criteria included: ① age < 60 years; ② ASA > IV; ③ patients who were intubated upon arrival in the operating room or undergoing a second surgery; ④ intracerebral hemorrhage caused by traumatic or space-occupying lesions; ⑤ presence of severe complications such as intracranial infection, coagulopathy, or severe dysfunction of vital organs like heart and lungs; ⑥ death or withdrawal from the study due to personal reasons during the study period.

Patients were randomly assigned to three groups using a random number table: control group (A), ropivacaine scalp nerve block group (B), and dexmedetomidine-ropivacaine scalp nerve block group (C), with 40 patients in each group. The study was approved by the Ethics Committee of Longyan First Affiliated Hospital of Fujian Medical University Hospital, and all participants provided written informed consent.

To determine the appropriate sample size, an analysis was conducted based on the primary endpoint, the VAS score for pain at 12 h postoperatively. The calculation was performed using PASS 12.0 software for a one-way ANOVA design comparing three groups, with a significance level (*α*) of 0.05 and a power (1-*β*) of 80%. The expected standard deviation (SD) was 6.9 mm, derived from pre-experimental data. A minimally clinically important difference (MCID) of 10 mm on the VAS was defined as the target effect size. The assumed mean VAS scores for the calculation was 45.0 mm for the control group, 35.0 mm for the ropivacaine SNB group (reflecting the MCID), and 30.0 mm for the dexmedetomidine-ropivacaine SNB group. Based on these parameters, the calculated minimum sample size was 37 patients per group. To account for potential dropouts, the final sample size was increased to 40 patients per group.

### Randomization and blinding

This study utilized a computer-generated random number table (SPSS 26.0 software) for patient randomization. The random sequence was generated by an independent statistician not involved in patient enrollment, intervention delivery, or outcome assessment. After generation, the sequence was stored in an encrypted electronic file accessible only to that statistician to ensure its integrity and unpredictability. Allocation concealment was achieved using sequentially numbered, opaque, sealed envelopes that were identical in appearance. These envelopes were prepared in advance by the same independent statistician according to the random sequence and stored in numerical order. Eligible patients, after providing informed consent, were assigned to the control group, ropivacaine SNB group, or dexmedetomidine-ropivacaine SNB group by a dedicated nurse who was unaware of the random sequence. The nurse opened the corresponding envelope in the order of patient enrollment to reveal the assignment, thus maintaining concealment.

A single-blinding strategy was employed. Due to the distinct nature of the interventions, the performing anesthesiologist could not be blinded. However, patients were blinded as the SNB was performed after anesthesia induction while they were unconscious. To minimize performance bias, a standardized intraoperative management protocol was established, including uniform protocols for anesthesia induction and maintenance, hemodynamic management targets, and criteria for initiating postoperative analgesia. All anesthesiologists were required to adhere strictly to this protocol, with deviations permitted only for predefined specific clinical scenarios as outlined in the protocol. Outcome assessors and the data-analyzing statistician remained blinded to group allocation. Unblinding was permitted solely in the event of a serious adverse event, requiring documentation and reporting; no such events occurred during the study.

### Anesthetic protocol

All patients were routinely fasted preoperatively and received no premedication. Upon arrival in the operating room, a peripheral intravenous line was established, and standard monitoring was initiated, including pulse oximetry (SpO_2_), heart rate (HR), non-invasive blood pressure (NIBP), electrocardiogram (ECG), and bispectral index (BIS). Under local anesthesia, an arterial line was inserted into the dorsalis pedis artery to measure invasive blood pressure, and an ultrasound-guided right internal jugular vein catheter was placed. Anesthesia induction was performed with propofol (Fresenius Kabi, Beijing, China; 20 mL: 0.2 g, Approval No.: J20160089) 2 mg/kg, sufentanil (Yichang Humanwell Pharmaceutical Co., Ltd., China; 1 mL: 50 μg, Approval No.: H20054171) 0.4 μg/kg, midazolam (Jiangsu Enhua Pharmaceutical Co., Ltd., China; 2 mL: 10 mg, Approval No.: H10980025) 0.05 mg/kg, and rocuronium bromide (Jiangsu Hengrui Medicine Co., Ltd., China; 5 mL: 10 mg, Approval No.: H20183042) 0.6 mg/kg. Endotracheal intubation was performed when BIS values decreased to 45–55. After intubation, patients were connected to an anesthesia machine, and mechanical ventilation was adjusted to maintain end-tidal carbon dioxide pressure (PetCO_2_) between 35 and 45 mmHg. Anesthesia was maintained with target-controlled infusion of propofol (Fresenius Kabi, Beijing, China; 50 mL: 500 mg, Approval No.: J20150661) and remifentanil (Yichang Humanwell Pharmaceutical Co., Ltd., China; 1 mg, Approval No.: H20171275), with propofol plasma target concentration set at 2.5–4.0 μg/mL and remifentanil plasma target concentration at 3–5 ng/mL. Adjustments were made based on BIS values and hemodynamic changes to maintain BIS between 45 and 55, and rocuronium bromide was intermittently used for muscle relaxation. Patients were transferred to the post-anesthesia care unit (PACU) with a tracheal tube in place and extubated when they met extubation criteria. Rescue analgesia was administered with intravenous flurbiprofen axetil (Beijing Ted Pharmaceutical Co., Ltd., China; 5 mL: 50 mg, Approval No.: H20041508) 1 mg/kg when postoperative visual analog scale (VAS) scores were ≥4. All surgeries were performed by the same team of surgeons.

### Nerve block technique

Due to the superficial yet variable anatomical location of scalp nerves and their proximity to major blood vessels, ultrasound guidance enables direct visualization of nerves, blood vessels, needle trajectory, and local anesthetic spread. This represents a shift from “blind” puncture to “visualized” technique, ensuring accurate identification of the target blockade site, avoiding intravascular injection, and improving the success rate and safety of the block. The specific procedural steps are as follows: with the patient in the supine position, standard skin disinfection and draping are performed. A high-frequency linear array probe is used for scanning. Under sterile conditions, the probe is placed over the anatomical surface projection area of the target nerve. Using a short-axis in-plane needle insertion technique, the needle tip is guided under real-time ultrasound imaging to the fascial plane surrounding the target nerve. After confirming the needle tip position and ensuring negative aspiration for blood, the local anesthetic is injected slowly while observing its spread around the target nerve.

Following anesthesia induction, ultrasound-guided SNB were performed according to the surgical incision site. Group B received selective blockade of the supraorbital, supratrochlear, zygomaticotemporal, auriculotemporal, greater occipital, and lesser occipital nerves using 0.5% ropivacaine (AstraZeneca AB, Sweden; 10 mL: 100 mg, Approval No.: H20140763). Group C received a mixture of 0.5% ropivacaine and 0.5 μg/kg dexmedetomidine (Jiangsu Hengrui Medicine Co., Ltd., China; 2 mL: 200 μg, Approval No.: H20090248) for selective blockade of the aforementioned nerves. Standard disinfection and draping were performed. The specific techniques were as follows: ① supraorbital nerve block: approximately 2 mL of local anesthetic was injected at the supraorbital notch; ② supratrochlear nerve block: approximately 2 mL of local anesthetic was injected at the intersection of the nasal root and the brow; ③ auriculotemporal nerve block: approximately 4 mL of local anesthetic was injected behind the temporomandibular pulse, 1.5 cm anterior to the tragus; ④ zygomaticotemporal nerve block: approximately 4 mL of local anesthetic was injected at the intersection of a line between the tragus and the supraorbital notch with the zygomatic bone; ⑤ greater occipital nerve block: approximately 4 mL of local anesthetic was injected at the lateral one-third of the line between the mastoid process and the external occipital protuberance; ⑥ lesser occipital nerve block: approximately 4 mL of local anesthetic was injected 2.5 cm lateral to the intersection of the nuchal line and the greater occipital nerve block site. All anesthetic procedures were performed by the same senior anesthesiologist.

### Outcome measures

The following parameters were compared among the three groups: ① mean arterial pressure (MAP) and heart rate (HR) at different time points during surgery (5 min before anesthesia induction-T1, 5 min after intubation-T2, incision-T3, dural incision-T4, and end of surgery-T5); ② intraoperative propofol and remifentanil consumption, and the time and number of postoperative rescue analgesic administrations; ③ VAS scores at 2, 12, 24, and 48 h postoperatively, where 0 indicates no pain, 1–3 indicates mild pain, 4–6 indicates moderate pain, and 7–10 indicates severe pain; ④ incidence of postoperative agitation and other adverse reactions within 48 h postoperatively.

### Statistical analysis

Data were analyzed using SPSS 26.0 software. Quantitative data with normal distribution are presented as mean ± standard deviation. Comparisons among the three groups were performed using one-way analysis of variance (ANOVA), followed by the Bonferroni *post-hoc* test for pairwise comparisons if the overall *p* value was significant. Categorical data are presented as numbers (percentages) and were analyzed using the chi-square test or Fisher’s exact test, as appropriate. A *p*-value of less than 0.05 was considered statistically significant.

## Results

### Comparison of general information

**Figure 1:**
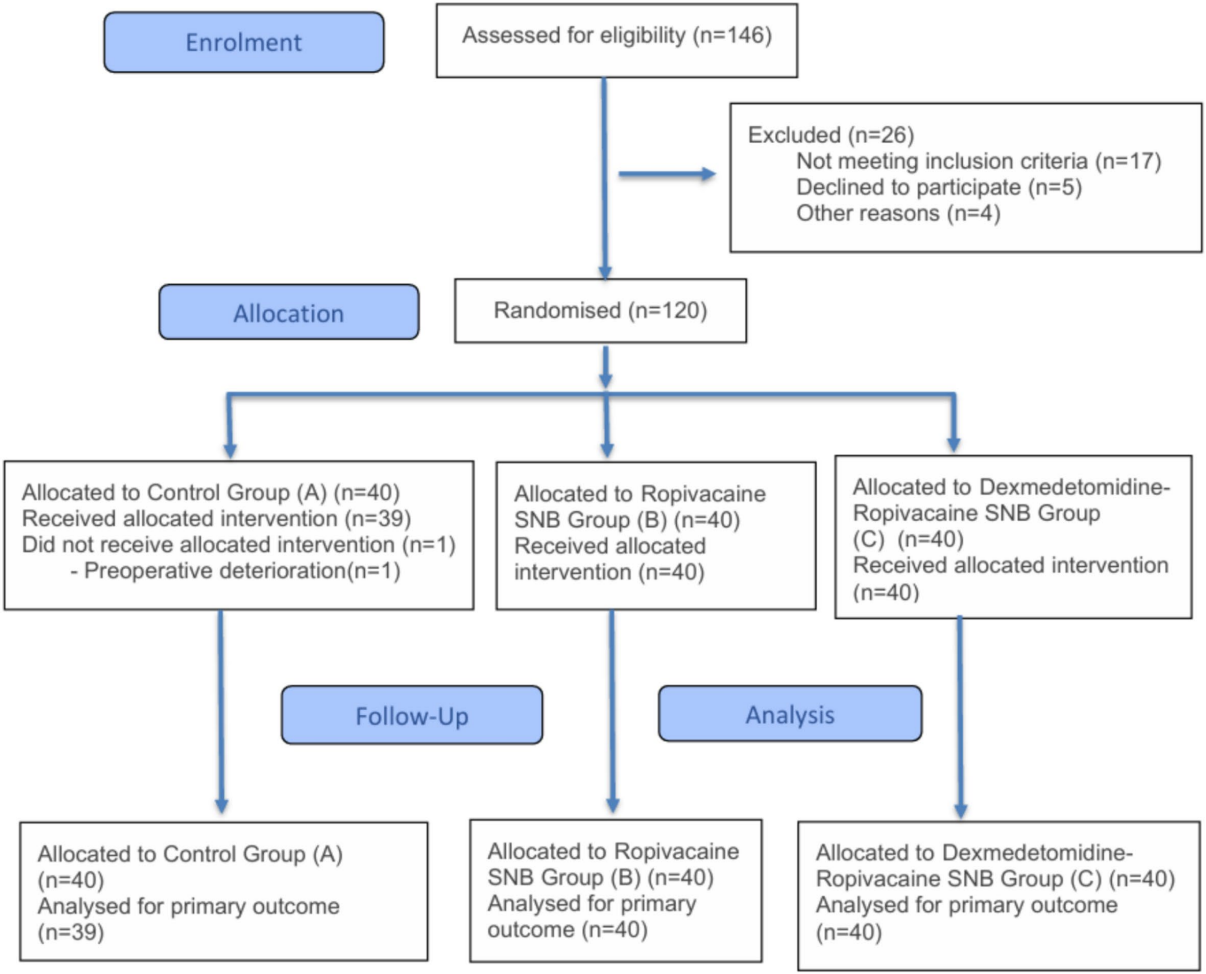
Flowchart of the study population.

The general characteristics of the three groups were compared, and no significant differences were observed (*p* > 0.05), as detailed in Table 1. The demographic data, including gender distribution, age, weight, ASA classification, and surgery time, were statistically similar across the control group, ropivacaine SNB group, and dexmedetomidine-ropivacaine SNB group, indicating that the groups were well-matched for these variables. Figure 1 shows the flowchart of the study population, and the specific values for each group are presented in Table 1.

**Table 1:** Comparison of general information among three groups.

Group	Gender (M/F)	Age (years)	Weight (kg)	ASA classification I/II (n)	Surgery time (min)
A	22/18	68.5 ± 6.5	62.0 ± 4.5	20/20	230.2 ± 21.1
B	23/17	69.0 ± 6.0	62.5 ± 4.0	21/19	232.5 ± 19.5
C	24/16	70.0 ± 5.5	63.0 ± 3.5	19/21	235.2 ± 18.9
*F*/*χ*^2^	0.178	0.456	0.544	0.211	0.432
*p*	0.915	0.635	0.582	0.900	0.668

The control group (A), ropivacaine SNB group (B), dexmedetomidine-ropivacaine SNB group (C). Data are presented as mean ± SD or number of patients (*n* = 40). Gender and ASA classification were compared using the chi-square test. Age, weight, and surgery time were compared using ANOVA.

### Hemodynamic stability

**Figure 2:**
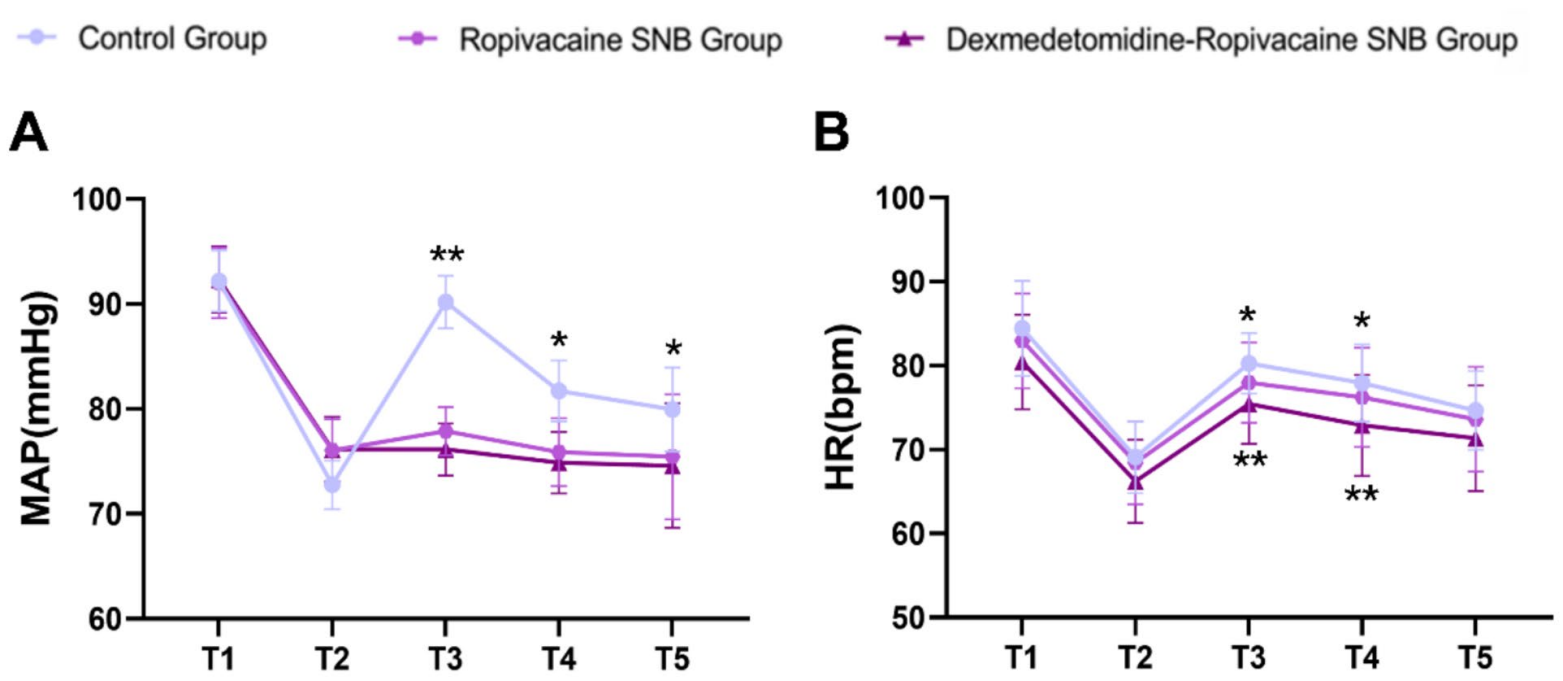
Changes in mean MAP **(A)** and HR **(B)**. T1, before induction; T2, after intubation; T3, at incision; T4, at dural incision; T5, end of surgery. Data were analyzed by repeated-measures ANOVA. ^**^p < 0.01, ^*^p < 0.05. The upper symbols are labeled as the comparison between Group A and Group B. The lower symbols are labeled as the comparison between Group A and Group C.

Hemodynamic changes are presented in Figure 2. At T3, T4, and T5, both the ropivacaine and dexmedetomidine-ropivacaine SNB groups showed significantly lower MAP compared to the control group (*p* < 0.01 at T3; *p* < 0.05 at T4 and T5). No significant difference in MAP was observed between the two nerve block groups at any time point (Figure 2A). These results indicate that the administration of ropivacaine and dexmedetomidine-ropivacaine for SNB may influence the hemodynamic stability, as reflected by the average arterial pressure, compared to the control group.

Regarding heart rate (Figure 2B), significant differences were found among the three groups at T3 and T4. *Post-hoc* analysis revealed that both nerve block groups had a significantly lower HR than the control group at these time points (*p* < 0.05). Furthermore, the dexmedetomidine-ropivacaine SNB group exhibited a significantly lower HR compared to the ropivacaine SNB group at T3 and T4 (*p* < 0.01) (Table 2). These findings indicate that the administration of ropivacaine and dexmedetomidine-ropivacaine for SNB may have a significant effect on HR, leading to a more pronounced decrease in HR compared to the control group.

**Table 2:** Comparison of MPA and HR among the three groups at different time points.

Parameter	Group	T1	T2	T3	T4	T5
HR (bpm)	A	84.6 ± 5.7	69.3 ± 4.8	81.9 ± 5.9	79.7 ± 6.5	75.4 ± 5.4
	B	82.8 ± 7.1	71.7 ± 6.0	76.0 ± 5.3	74.2 ± 5.4	74.6 ± 6.4
	C	80.5 ± 7.0	70.4 ± 6.3	71.4 ± 5.2	70.0 ± 5.8	72.5 ± 6.5
MAP (mmHg)	A	92.5 ± 4.4	75.9 ± 3.3	88.4 ± 5.7	82.7 ± 4.3	80.6 ± 5.8
	B	91.6 ± 7.1	76.5 ± 3.4	77.0 ± 3.1	74.6 ± 4.1	76.6 ± 5.8
	C	91.7 ± 6.7	76.1 ± 3.4	75.9 ± 3.8	73.5 ± 4.1	73.6 ± 6.2

The control group (A, *n* = 39), ropivacaine SNB group (B, *n* = 40), dexmedetomidine-ropivacaine SNB group (C, *n* = 40). Data are presented as mean ± SD. HR, heart rate; MAP, mean arterial pressure.

### Postoperative pain assessment

**Figure 3:**
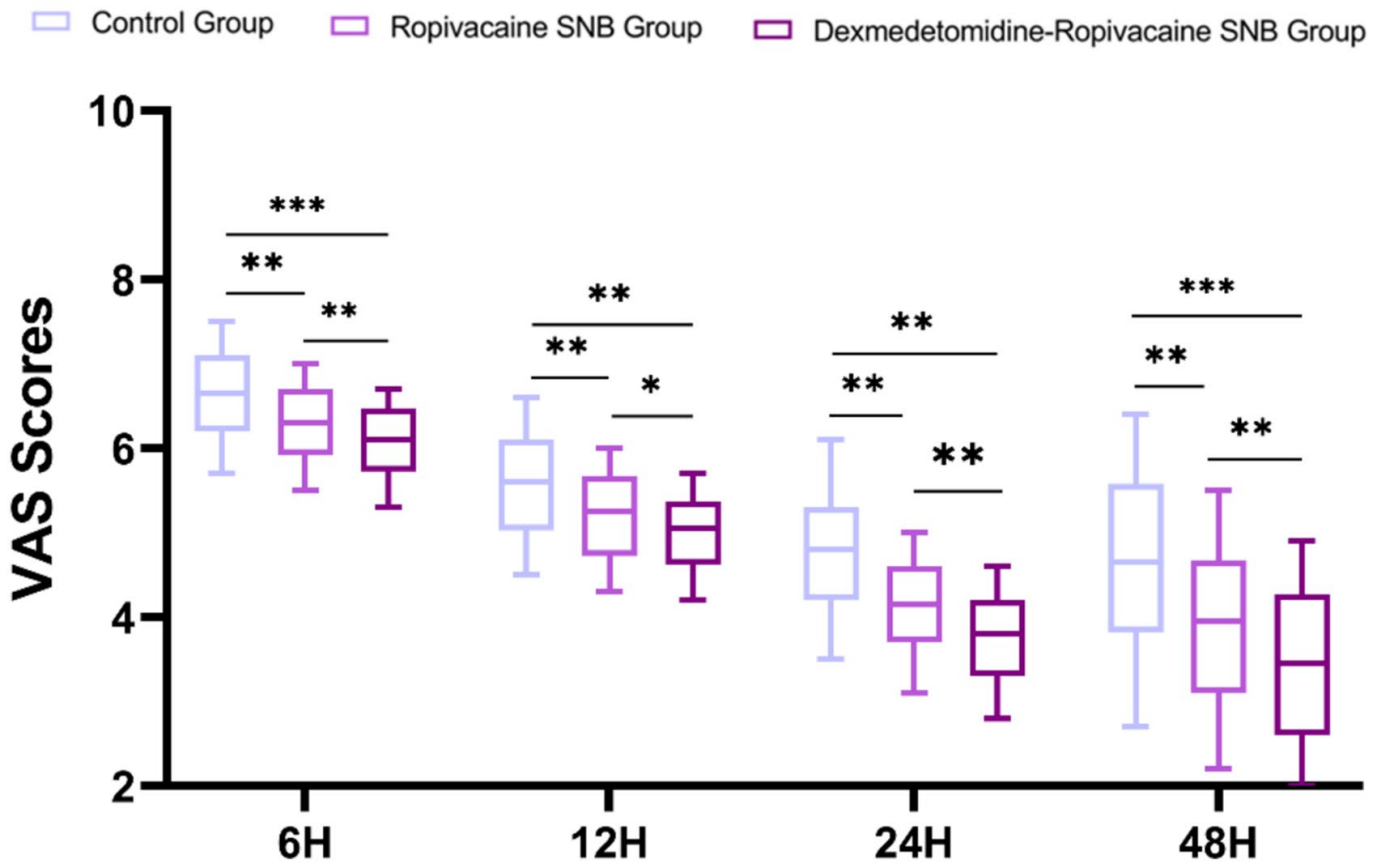
Differences in VAS scores at different times between groups (scores). Data were analyzed by repeated-measures ANOVA. ^*^p < 0.05, ^**^p < 0.01, ^***^p < 0.001.

Postoperative pain was evaluated using the VAS at 6, 12, 24, and 48 h after surgery to compare the analgesic effects among the three groups (Figure 3). Before the VAS score assessment, the Glasgow Coma Scale (GCS) had been used to evaluate the patients’ consciousness status, and all GCS scores were greater than 14. Compared with the control group, the ropivacaine SNB and dexmedetomidine-ropivacaine SNB groups showed significantly lower VAS scores at 6, 12, 24, and 48 h postoperatively (*p* < 0.05). Furthermore, the dexmedetomidine-ropivacaine SNB group demonstrated significantly lower VAS scores than the ropivacaine SNB group from 6 to 48 h after surgery. These result suggest that the addition of dexmedetomidine to ropivacaine for SNB provides superior pain control in the early postoperative period compared to ropivacaine alone or no nerve block.

### Anesthetic usage and postoperative analgesia

**Figure 4:**
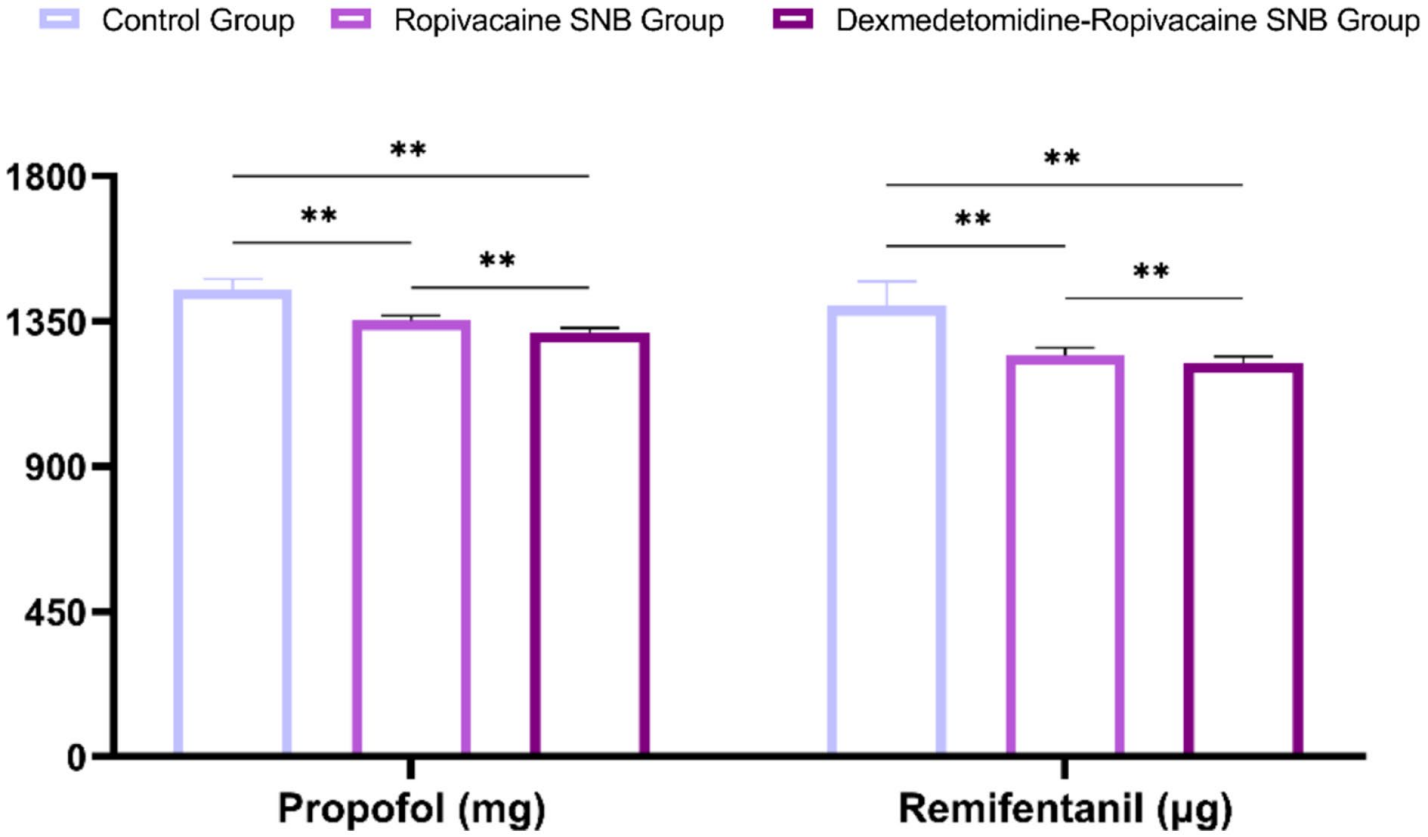
Comparison of intraoperative propofol and remifentanil consumption.

As summarized in Table 3, SNB significantly reduced intraoperative anesthetic requirements and improved postoperative analgesia. Both the ropivacaine and dexmedetomidine-ropivacaine SNB group demonstrated lower consumption of propofol and remifentanil compared to the control group (Figure 4) (*p* < 0.05). Correspondingly, the need for first rescue analgesia was substantially delayed, and its frequency within 48 h was markedly reduced in the nerve block groups (*p* < 0.05). Notably, the dexmedetomidine-ropivacaine SNB group showed superior outcomes compared to the ropivacaine SNB group across all parameters (*p* < 0.05).

**Table 3:** Intergroup comparison of anesthetic and analgesic outcomes.

Group	Propofol (mg)	Remifentanil (μg)	First rescue analgesia time (min)	Rescue analgesia frequency (times)
A	1451.6 ± 126.4	1376.8 ± 148.0	486.9 ± 67.4	1.41 ± 0.8
B	1353.5 ± 90.6	1249.7 ± 119.9	654.2 ± 70.3	0.85 ± 0.7
C	1315.1 ± 102.3	1226.3 ± 120.2	701.1 ± 75.3	0.53 ± 0.5
*t*-value	3.457	3.654	9.410	2.966
*p*	0.01	0.01	0.01	0.01

The control group (A, *n* = 39), ropivacaine SNB group (B, *n* = 40), dexmedetomidine-ropivacaine SNB group (C, *n* = 40). Data was presented as mean ± SD. Intergroup comparisons for all continuous variables were first analyzed using ANOVA. If the overall ANOVA indicated a significant difference, *post-hoc* pairwise comparisons were then performed using the Bonferroni correction. a, *p* < 0.05 compared with Group A; b, *p* < 0.05 compared with Group B.

### Postoperative adverse reactions at 48 h

The incidences of key postoperative adverse reactions within 48 h are summarized in Table 4. The rate of nausea/vomiting was significantly lower in both the Ropivacaine (10.0%) and dexmedetomidine-ropivacaine (5.0%) SNB groups compared to the control group (25.0%, *p* < 0.05). A similar trend was observed for postoperative agitation, with incidences of 27.5% in the control group, compared to 10.0 and 7.5% in the respective SNB groups (*p* < 0.05 vs. control).

**Table 4:** Incidence of postoperative adverse reactions within 48 h.

Adverse reaction	Group A	Group B	Group C	*p*
Nausea/vomiting	10 (25.0%)	4 (10.0%)	2 (5.0%)	0.021
Postoperative agitation	11 (27.5%)	4 (10.0%)	3 (7.5%)	0.015
Hematoma	1 (2.5%)	0	1 (2.5%)	0.599

The control group (A, *n* = 39), ropivacaine SNB group (B, *n* = 40), dexmedetomidine-ropivacaine SNB group (C, *n* = 40). Data was presented as mean ± SD. Intergroup comparisons for all continuous variables were first analyzed using ANOVA. If the overall ANOVA indicated a significant difference, *post-hoc* pairwise comparisons were then performed using the Bonferroni correction.

Regarding block-related complications, small, self-limiting hematomas were observed in 1 patient (2.5%) in control and dexmedetomidine-ropivacaine SNB group, which resolved without intervention. No cases of local infection, respiratory depression, hypotension, re-bleeding or bradycardia event occurred in any group. Further *post-hoc* comparisons revealed no statistically significant difference in the incidence of any adverse event between the two nerve block groups (*p* > 0.05), indicating a favorable and comparable safety profile for both SNB regimens.

## Discussion

Hypertensive cerebral hemorrhage is a common emergency with high rates of disability and mortality (11). Surgery is a common treatment method, especially for patients with large hemorrhages, who require early craniotomy to effectively remove the hematoma, control intracranial pressure, and prevent brain herniation (12). Craniotomy carries significant surgical risks, and anesthesia must reduce hemodynamic fluctuations, lower surgical stress responses, especially maintain blood pressure stability, while ensuring cerebral perfusion and reducing the occurrence of postoperative agitation to ensure a smooth emergence from anesthesia (13). However, operations such as scalp incision and cranial drilling during craniotomy can cause significant stimulation to the nervous system, triggering increased blood pressure and heart rate, thereby increasing surgical risks and affecting the smooth progress of the surgery (14). Conventional general anesthesia, while providing good anesthetic effects, is not ideal for reducing stress responses and is not conducive to postoperative recovery. Therefore, effective analgesic measures are necessary.

Local anesthetics for preemptive analgesia are effective methods for postoperative pain control, and preoperative analgesia can prevent central sensitization caused by noxious stimuli and inflammation (15). Studies have shown that SNB with 0.2% ropivacaine provides stable analgesic effects, relieving postoperative pain for up to 3–8 h (6, 16, 17). Hemodynamic stability is crucial for neurosurgical patients both intraoperatively and postoperatively. Elevated blood pressure can lead to a sudden increase in intracranial pressure, causing uncontrollable bleeding. In the absence of regional anesthesia, deep anesthesia is often used to control hemodynamic changes in response to noxious stimuli, including increased concentrations of opioid and/or hypnotic anesthetics. In our study, we found that compared to the control group, the SNB group maintained stable hemodynamics during periods of intense noxious stimulation. Additionally, SNB can reduce surgical pain and aid in early postoperative recovery (18). Furthermore, the duration of scalp block anesthesia is relatively long, providing analgesic effects even 12 h postoperatively. Ropivacaine is a commonly used local anesthetic for SNB, with good analgesic effects and few adverse reactions, offering advantages over other anesthetics and is widely used in nerve block anesthesia (19). Studies have shown that 0.5% ropivacaine is more effective for surgical analgesia than 0.25% ropivacaine, significantly reducing postoperative pain (20). Due to the significant trauma of craniotomy, ropivacaine SNB can effectively reduce painful stimulation, maintain intraoperative hemodynamic stability, and reduce the occurrence of various complications. Our study found that the application of ropivacaine SNB helps to reduce fluctuations in intraoperative hemodynamic indicators, alleviate postoperative pain, and reduce the incidence of postoperative agitation, suggesting its clinical applicability.

Dexmedetomidine, a potent *α*_2_-adrenergic receptor agonist, possesses sedative, analgesic, anxiolytic, and antiemetic properties. When administered intravenously, it does not significantly suppress respiration and, when used as an adjuvant in peripheral nerve blocks, can shorten the onset time and extend the duration of analgesia (21). Brummett et al. (22) found that the addition of dexmedetomidine to ropivacaine for sciatic nerve block in rats extended the analgesic duration by approximately 75%, possibly by blocking the hyperpolarization-activated cation current. Marhofer et al. (23) reported that the addition of 20 μg dexmedetomidine to 0.75% ropivacaine for ultrasound-guided ulnar nerve block significantly extended the analgesic duration by about 60%, whereas intravenous administration of 20 μg dexmedetomidine only extended analgesia by about 10%. Sarotti et al. (24) found that dexmedetomidine significantly prolonged the duration of sensory block without extending motor block or increasing bradycardia events in dogs undergoing spinal anesthesia. Vallapu et al. (25) demonstrated that the addition of dexmedetomidine to bupivacaine for SNB significantly prolonged the analgesic duration in patients undergoing craniotomy, consistent with our findings. In the present study, postoperative VAS scores were found to be significantly reduced in both intervention groups compared to the control group at all measured time points (6, 12, 24, and 48 h). Superior analgesic efficacy was further demonstrated in the dexmedetomidine-ropivacaine SNB group, which exhibited significantly lower VAS scores from 6 to 48 h compared to the ropivacaine SNB group alone. This consistency with previous research confirms the significant enhancement in postoperative analgesia achieved by combining dexmedetomidine with ropivacaine for SNB.

To evaluate the efficacy of the combined dexmedetomidine-ropivacaine SNB, it was found that patients receiving this mixture required less general anesthetic during surgery, experienced better postoperative analgesia, and demonstrated a significantly delayed time to first rescue analgesia along with a significantly reduced frequency of rescue analgesic requirements within 48 h postoperatively, compared to both the control group and the group receiving ropivacaine alone. This may be due to the slow absorption of dexmedetomidine into the bloodstream, which activates peripheral nerve *α*_2_A receptors and blocks hyperpolarization-activated cation currents to produce analgesia. Group C had lower incidences of postoperative agitation and nausea/vomiting, possibly because dexmedetomidine acts on peripheral and central *α*_2_-adrenergic receptors, producing sedative effects (26). Dexmedetomidine may reduce the production of norepinephrine by acting on *α*_2_-adrenergic receptors on noradrenergic neurons in the locus coeruleus, thereby reducing nausea and vomiting (27). In conclusion, the addition of 0.5 μg/kg dexmedetomidine to ropivacaine for SNB is well-tolerated and associated with significant improvements in postoperative outcomes for patients undergoing craniotomy. The findings of this study directly address a gap in the current evidence base as highlighted by the recent meta-analysis (10). While that synthesis identified a lack of moderate-to-high certainty evidence regarding the effectiveness of adjuvant dexmedetomidine in scalp blocks, our results provide prospective RCT data demonstrating that the addition of dexmedetomidine to ropivacaine for SNB significantly prolongs analgesia, reduces opioid consumption, and improves hemodynamic stability compared to ropivacaine alone in patients undergoing craniotomy for hypertensive cerebral hemorrhage.

The safety profile observed in this trial was favorable. No events of respiratory depression, hypotension, or bradycardia were recorded in any group. However, it is crucial to interpret these safety outcomes with caution. While the sample size was adequate to detect differences in the primary analgesic endpoints, it remains underpowered to reliably assess the incidence of uncommon or rare adverse events. The favorable safety data presented here should therefore be considered preliminary in this context. Moreover, several limitations of this study merit acknowledgment. A primary constraint was the single-blind nature of the trial; despite efforts to blind data analysts, the administering anesthesiologists could not be blinded, which may have introduced a potential source of bias. The generalizability of our results is also tempered by the moderate sample size and the relatively short 48-h monitoring period, which preclude assessments of long-term outcomes. Additionally, the absence of dose gradients for dexmedetomidine means the optimal effective dose remains undefined. Future research should aim to address these limitations through larger, multi-center trials with extended follow-up and dose-ranging designs.

## Conclusion

This prospective randomized controlled study investigated the analgesic efficacy of dexmedetomidine combined with ropivacaine in ultrasound-guided SNB for patients with intracerebral hemorrhage. Compared with the non-SNB control group, the SNB groups showed improved postoperative analgesia, stable intraoperative hemodynamics, reduced consumption of anesthetics and requirements for rescue analgesia, and lower incidence of adverse reactions in patients undergoing craniotomy for HICH. Notably, the dexmedetomidine-ropivacaine group had significantly lower VAS scores and less anesthetic consumption than the ropivacaine-only group. In summary, SNB with dexmedetomidine combined with ropivacaine is a potentially safe and effective perioperative analgesic strategy. Future prospective multicenter studies with multi-dose gradient effects and long-term follow-up of multiple indicators are needed to identify the optimal dose combination and verify the universality and stability of this medication regimen.

## Data Availability

The original contributions presented in the study are included in the article/supplementary material, further inquiries can be directed to the corresponding author.
